# Construction and validation of a predictive nomogram for vascular crisis risk following free flap surgery

**DOI:** 10.3389/fmed.2026.1930369

**Published:** 2026-10-20

**Authors:** Yan-liang Qu, Juan Xue, Xing-yu Du, Dai-di Zhang, Yue Wang, Ya-lin Zhu, Jia-feng Wang

**Affiliations:** 1 Faculty of Anesthesiology, Changhai Hospital, Naval Medical University, Shanghai, China; 2 Department of Hand Surgery Anesthesiology, No. 971 Hospital of the People’s Liberation Army Navy, Qingdao, Shandong, China; 3 Department of Nursing, No. 971 Hospital of the People’s Liberation Army Navy, Qingdao, Shandong, China; 4 Department of Nephrology, No. 971 Hospital of the People’s Liberation Army Navy, Qingdao, Shandong, China; 5 Department of Anesthesiology, Qingdao University Affiliated Hospital, Qingdao, Shandong, China; 6 Department of Anesthesiology, Naval Medical Center, Naval Medical University, Shanghai, China

**Keywords:** free flap, nomogram, prediction, risk factors, vascular crisis

## Abstract

**Introduction:**

This study aimed to construct and validate a predictive nomogram for estimating the risk of postoperative vascular crisis in patients undergoing free flap surgery.

**Methods:**

Clinical medical records of 568 patients who underwent free flap surgery at No. 971 Hospital of the People's Liberation Army Navy between January 2012 and August 2023 were retrospectively reviewed. Based on the admission date, the training set comprised 402 patients, and the remaining 166 patients constituted the validation set. Multivariate logistic regression was used to select the variables. R software was used to develop the vascular crisis risk prediction model, evaluate its predictive performance, and visualize the model. Subsequently, the model was internally validated using the validation set.

**Results:**

Among 568 patients, the overall incidence of vascular crisis after free flap surgery was 11.1%. A nomogram was constructed using four independent risk factors: serum albumin level, smoking, hemoglobin level, and packed red blood cell suspension transfusion. The area under the receiver operating characteristic curve (AUC) was 0.817 (95% confidence interval [CI]: 0.747–0.887) for the training set and 0.852 (95% CI: 0.774–0.929) for the validation set. The calibration curve indicated satisfactory concordance between the nomogram-estimated risk and the actual clinical incidence, with a mean absolute error of 0.019 (<0.05). Decision curve analysis confirmed the favorable clinical application value of this nomogram.

**Discussion:**

The nomogram demonstrated appreciable predictive performance for vascular crisis risk in patients undergoing free flap surgery. This tool may assist in identifying high-risk populations and facilitate appropriate clinical decision-making.

**Clinical trial regidstration:**

https://www.chictr.org.cn/, China Clinical Trials Registry ChiCTR2400087348

## 1 Introduction

Free flap transplantation is a vital microsurgical technique widely used for repairing severe soft tissue defects and reconstructing limb and external organ functions, particularly in cases of trauma or cancer resection (1). With continuous advances in microsurgical anastomosis techniques, surgical instruments, and surgical robots, the quality of vascular anastomoses has significantly improved. Nevertheless, postoperative vascular crises (i.e., thrombosis or spasm of the anastomosed vessels) remain the leading perioperative complication, causing flap necrosis and surgical failure. Vascular crises often necessitate emergent salvage surgery, significantly prolong hospital stays, increase patients’ financial burden, and may even lead to permanent impairment of limb function (1–3).

The overall failure rate of free flap surgery ranges from 5% to 10%, whereas the incidence of vascular crisis is between 5% and 25% (4, 5). These outcomes are affected by multiple factors, including baseline patient conditions, surgical site, flap type, and perioperative management quality (6). Currently, clinical strategies for preventing and managing vascular crises are largely based on passive postoperative monitoring, with interventions initiated only after the appearance of typical signs of flap ischemia or congestion (7). However, early vascular crises present subtle manifestations that may go unrecognized. Moreover, once irreversible microcirculatory damage occurs, the success rate of salvage surgery decreases sharply from >80% to <30% (8). Therefore, there is an urgent need to establish an early risk warning system that enables a shift from passive to active prevention and control (9).

In recent years, local and international researchers have identified preliminary risk factors for vascular crises in free flaps based on available evidence, including patient age, chronic conditions, smoking history, operative duration, and anastomosis technique (10, 11). However, the existing studies have notable limitations. First, most previous models have largely focused on preoperative baseline characteristics and surgical factors while generally overlooking key modifiable perioperative variables, such as anesthetic management, perioperative volume status, oxygenation, and analgesic levels (12, 13). Second, most current models provide static risk assessments and lack user-friendly visualization tools. Furthermore, they do not provide rapid bedside clinical decision-making guidance. Therefore, a key challenge for future research is to integrate multidimensional risk factors and develop precise predictive models to guide personalized perioperative management. The goal is to establish a proactive risk management strategy spanning from prediction to intervention, eventually reducing the incidence of vascular crises.

The nomogram model, based on multivariate logistic regression, provides intuitive results and facilitates rapid bedside assessment, which better aligns with frontline clinical decision-making. Therefore, it is widely used to predict the risk of surgical and perioperative complications (14, 15). In terms of pathogenesis, vascular crisis results primarily from an imbalance caused by the interaction between the patient's general condition, surgical trauma stress, and perioperative anesthetic management (16–18). Anesthesia-related disturbances, including hypotension, hypothermia, inadequate analgesia, and inappropriate vasopressor use, can directly impair the microcirculatory stability of free flaps and serve as core modifiable targets for managing the risk of vascular crises (19, 20). However, current vascular crisis prediction models rarely integrate preoperative baseline status and perioperative anesthetic parameters. Consequently, they lack quantitative support for postoperative screening of high-risk patients, intraoperative personalized anesthetic management, and postoperative intensive monitoring, and fail to meet the clinical requirements of precision medicine during the perioperative period.

To address these research gaps, this retrospective investigation included analysis of clinical records of patients who underwent free flap transplantation at our hospital over the past 12 years. Independent predictors of perioperative vascular crisis were identified, and a visualized nomogram risk assessment model was constructed and internally validated. This study aimed to provide evidence-based guidance for refined perioperative management, enabling the early postoperative detection of at-risk individuals and facilitating individualized interventions in free flap surgery.

## 2 Materials and methods

### 2.1 Study population

This single-center, retrospective study was conducted at the No. 971 Hospital of the People's Liberation Army Navy. Information was retrieved from the clinical records of individuals who underwent free flap procedures between January 2012 and August 2023. A total of 568 patients were included in this study.

This study was conducted in accordance with the principles of the Declaration of Helsinki (2024). The study was reviewed and approved by the Medical Ethics Committee of the No. 971 Hospital of the People's Liberation Army Navy (approval no.: 971LL-20231207001) and registered with the China Clinical Trials Registry at https://www.chictr.org.cn/ (trial no.: ChiCTR2400087348). The Ethics Committee waived the requirement for written informed consent because the data did not contain any personally identifiable information. This study is reported in accordance with the STROBE (Strengthening the Reporting of Observational Studies in Epidemiology) guidelines. The completed STROBE and TRIPOD checklists are provided as Supplementary files.

### 2.2 Inclusion and exclusion criteria

Initially, 1,084 patient records were extracted and manually screened to identify the relevant surgical information. The inclusion criteria were as follows: 1) age ≥6 (the lower limit was set based on considerations regarding the technical feasibility of microvascular anastomosis and perioperative management in pediatric patients) (21), 2) any sex, and 3) patients undergoing free flap surgery. The exclusion criteria were as follows: 1) age <6 years, 2) ASA Physical Status Class IV or V, 3) patients undergoing non-free flap surgery, and 4) patients who underwent but failed to complete free flap surgery.

### 2.3 Outcome measures

The primary endpoint was the occurrence of postoperative vascular crises. The diagnosis of vascular crisis was based on comprehensive bedside clinical monitoring, which remains the gold standard for assessing free flap viability. The following five core parameters were routinely evaluated: flap color, flap temperature, capillary refill time, flap tension, and bleeding upon pinprick. Arterial crisis was suspected when the flap appeared pale or grayish, with decreased temperature, prolonged or absent capillary refill, decreased turgor with deepening of the skin folds, and scant or absent bleeding upon pinprick. Venous crisis was suspected when the flap appeared dark red or cyanotic, with decreased temperature, shortened or rapid capillary refill, increased turgor with swelling and tension, and profuse dark-red bleeding upon pinprick (22, 23). All cases were independently assessed and confirmed by two senior microsurgeons to ensure diagnostic objectivity and consistency. Discrepancies were resolved through discussion or by involving another senior microsurgeon.

### 2.4 Data collection

Data for this study were obtained from the Information and Quality Management Departments of the hospital. The Data Division identified target patients by searching for surgical procedure names, and the researchers retrieved complete electronic medical records from the Quality Management Department. All data were independently extracted by two investigators. Discrepancies were resolved through discussion or by involving another senior investigator.

In line with the established research framework and current state of medical records, the individual data extracted from previous medical records primarily included the following categories: demographic indicators, preoperative baseline characteristics, preoperative laboratory results, intraoperative surgical and anesthetic parameters, and postoperative outcomes.

Demographic indicators included basic patient information, such as age, sex, body temperature, pulse, respiration, and weight.

The preoperative baseline characteristics included the American Society of Anesthesiologists (ASA) physical status classification, hypertension, diabetes, cardiac comorbidities, chronic kidney disease, pulmonary comorbidities, liver disease, smoking history, and alcohol consumption history.

The preoperative laboratory results included hemoglobin (Hb), hematocrit, platelet count, serum albumin (ALB), fasting blood glucose, blood urea nitrogen, creatinine, aspartate aminotransferase (AST), alanine aminotransferase (ALT), prothrombin time (PT), and activated partial thromboplastin time (APTT).

The intraoperative surgical and anesthetic parameters included operative time, blood loss, SpO_2_, volume of crystalloid infusion, volume of colloid infusion, intraoperative fluid balance, intraoperative urine output, volume of packed red blood cell (PRBC) intraoperative transfusion, volume of plasma transfusion, intake of diuretic medications and nonsteroidal anti-inflammatory agents, glucocorticoid use, and vasoactive agent use.

The postoperative outcomes included vascular crisis and flap survival.

### 2.5 Perioperative management

All 568 patients who underwent free flap surgery received a standardized postoperative anticoagulation regimen. Specifically, low-molecular-weight heparin sodium was administered by subcutaneous injection into the abdominal wall, and intramuscular injection was prohibited. The standard dose was 4,000 anti-Xa IU (40 mg) once daily, continued for the first 7 postoperative days. This anticoagulation regimen is consistent with commonly reported regimens in the literature (24).

The use of vasoactive agents was managed according to the standardized perioperative management protocol for free flap surgery. Specifically, vasoactive agents (phenylephrine) were administered only under the following conditions: (1) persistent intraoperative hypotension with systolic blood pressure < 90 mmHg or mean arterial pressure < 65 mmHg that could not be corrected despite adequate fluid resuscitation; or (2) severe hypotension occurring after induction of anesthesia. Phenylephrine was administered as an intravenous bolus of 40–100 μg with repeat doses given as clinically indicated.

As all patients received the same two regimens, there were no between-patient differences in postoperative anticoagulation or the use of vasoactive medication.

### 2.6 Handling of missing data

Missing data were assessed for all candidate predictors and outcome variables. Missingness was assumed to be missing at random based on the assessment that missing values were primarily related to clinical workflow rather than to the unobserved laboratory values themselves.

Missing data were handled separately in the training and validation cohorts. For continuous variables, missing values were imputed using the mean for variables with a normal distribution and the median for variables with a non-normal distribution. For categorical variables, missing values were imputed using the mode. Finally, for continuous variables with an approximately normal distribution, missing values were imputed using the variable-specific overall mean within the training and validation cohorts. To assess the robustness of the primary analysis to the missing data handling approach, a complete-case analysis was additionally performed as a sensitivity analysis.

### 2.7 Statistical analysis

Statistical analyses were performed using SPSS software (version 25.0; IBM Corp., Chicago, IL, USA). A skewness-kurtosis approach was applied to test for normality of distribution of the continuous variables. Continuous variables with a Gaussian distribution are presented as mean ± standard deviation, whereas those without a normal distribution are summarized as medians with interquartile ranges (IQR). Categorical parameters are expressed as frequencies (percentages).

Using December 31, 2020, as the cutoff point, all 568 patients were stratified into two subgroups. Cases between January 1, 2012, and December 31, 2020, were assigned to the training cohort (*n* = 402), whereas cases from January 1, 2021, to August 30, 2023, served as the validation cohort (*n* = 166). Patients in the training cohort were used to construct a predictive tool for the risk of vascular crisis in free flaps.

First, a univariate analysis was performed in the training set using postoperative vascular crisis as the endpoint. Continuous variables were compared using the Mann–Whitney U test, whereas categorical variables were analyzed using the chi-square or Fisher's exact test. Second, variables with a P-value < 0.10 in the univariate analysis were included in the multivariate logistic regression model. The sample size was estimated following the events per variable principle; each predictor was supported by at least 10 endpoint events to ensure the stability of the regression coefficient estimation (25). Finally, the independent predictors of vascular crisis risk were identified using stepwise regression.

R 4.0.1 (R Foundation for Statistical Computing, Vienna, Austria) was used to establish the risk prediction model and plot the nomogram. Patient data from the validation cohort were used for internal validation of the risk prediction nomogram. A receiver operating characteristic (ROC) curve was plotted and the area under the ROC curve (AUC) was calculated to assess the discriminatory power of the model. Bootstrap resampling was implemented to examine model calibration, and decision curve analysis (DCA) was used to quantify the model's clinical practicability. Statistical significance was defined as a *P*-value of < 0.05.

## 3 Results

### 3.1 Demographic and clinical characteristics

From January 2012 to August 2023, a pool of 1,084 qualified individuals was initially screened by searching with the keyword ‘flap’ on the medical record homepages of all hand surgeries. We excluded 500 patients who underwent pedicle or locally mobilized flap surgery (non-free flap surgery) because the type of surgery did not meet the inclusion criteria, or because of coding errors. Among the remaining 584 patients, one did not undergo free flap surgery because of inability to identify perforating vessels, and another did not meet the criteria for free flap surgery, which was then converted to a pedicle flap procedure. In addition, 14 patients were aged < 6 years. In total, 568 patients were included in this study (Figure 1). Patients were allocated to a training set (402 patients, including 42 with vascular crises) or a validation set (166 patients, including 21 with vascular crises). The median age of the study population was 44 (IQR, 31–51) years. The age range of the included patients was 6–73 years. In total, 13 (2.3%) patients had a medical history of hypertension, three (0.5%) had cardiovascular disease, 12 (2.1%) had diabetes, and one (0.2%) had chronic kidney disease. The median operative duration was 7 h (IQR, 5.5–8.5). Table 1 shows the demographic and clinical characteristics of the study participants.

**Figure 1:**
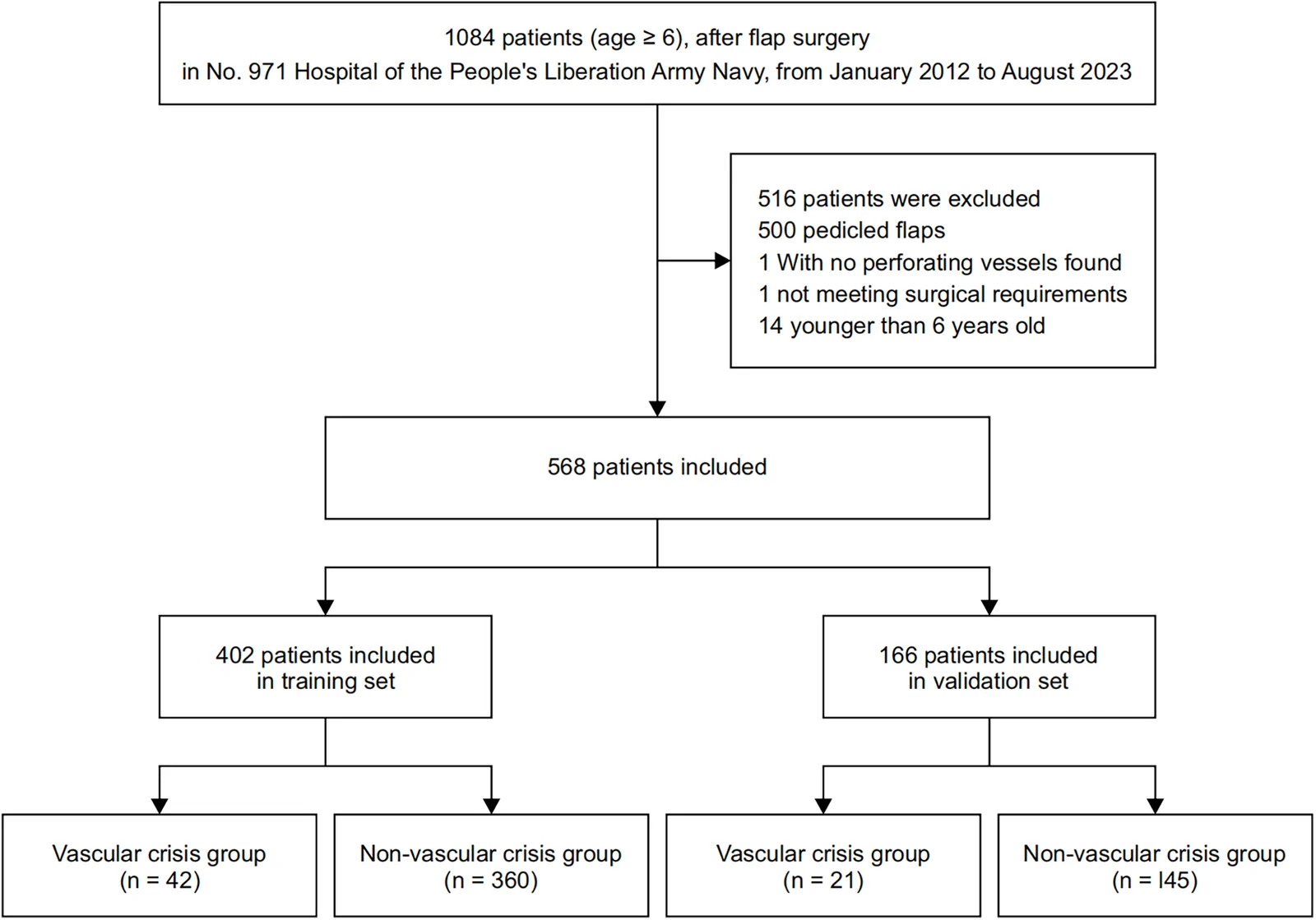
Flowchart of patient enrolment and grouping into training and validation sets.

**Table 1:** Demographic and clinical variables of patients in the training and validation sets.

Variables	All (*n* = 568)	Training (*n* = 402)	Validation (*n* = 166)	*p*-value
Age, years, M (P25, P75)	44 (31–51)	42.5 (29–49)	47 (39–54)	<0.001
Sex (Male), *n* (%)	456 (80.28)	324 (80.60)	132 (79.52)	0.769
Weight, kg, M (P25, P75)	65 (60–75)	65 (60–75)	70 (60–75)	0.001
Pulse, beats per min, M (P25, P75)	79 (71–82)	80 (72–85)	78 (70–80)	0.009
Respiration, breaths per min, M (P25, P75)	16 (16–18)	16 (16–18)	16 (16–18)	0.427
SBP, mmHg, M (P25, P75)	130 (120–139)	128 (120–135)	130 (120–140)	0.014
DBP, mmHg, M (P25, P75)	80 (73–89)	80 (70–85)	83 (76–90)	<0.001
Temperature, ℃, M (P25, P75)	36.5 (36.5–36.6)	36.5 (36.5–36.7)	36.5 (36.4–36.5)	<0.001
ASA PS, *n* (%)				0.359
Ⅰ	337 (59.33)	247 (61.44)	90 (54.22)	
Ⅱ	217 (38.20)	142 (35.32)	75 (45.18)	
Ⅲ	14 (2.46)	13 (3.23)	1 (0.60)	
Peripheral vascular disease, *n* (%)	0 (0.00)	0 (0.00)	0 (0.00)	1.000
Hypertension, *n* (%)	13 (2.29)	7 (1.74)	6 (3.61)	0.294
Cardiovascular diseases, *n* (%)	3 (0.53)	2 (0.50)	1 (0.60)	1.000
Diabetes, *n* (%)	12 (2.11)	5 (1.24)	7 (4.22)	0.055
Chronic kidney disease, *n* (%)	1 (0.18)	0 (0.00)	1 (0.60)	0.293
Pulmonary diseases, *n* (%)	1 (0.18)	1 (0.25)	0 (0.00)	1.000
Liver disease, *n* (%)	2 (0.35)	2 (0.50)	0 (0.00)	1.000
Smoking, *n* (%)	226 (39.79)	165 (41.04)	61 (36.75)	0.341
Alcohol, *n* (%)	198 (34.86)	133 (33.08)	65 (39.16)	0.167
Hemoglobin, g/L, M (P25, P75)	128 (111–142)	128 (110–144)	128 (113–139)	0.991
Hematocrit, %, M (P25, P75)	38.5 (33.0–42.5)	38.3 (32.2–42.4)	39.0 (34.8–42.6)	0.075
Platelets, 10⁹/L, M (P25, P75)	250 (207–312)	248 (206–311)	259 (210–321)	0.200
ALB, g/L, M (P25, P75)	37.2 (33.4–41.6)	37.1 (32.7–41.1)	37.2 (34.3–42.7)	0.038
Fasting blood glucose, mmol/L, M (P25, P75)	5.2 (4.7–5.8)	5.2 (4.7–5.9)	5.3 (4.9–5.7)	0.243
Blood urea, mmol/L, M (P25, P75)	4.5 (3.6–5.5)	4.4 (3.5–5.4)	4.9 (3.9–5.8)	<0.001
Creatinine, μmol/L, M (P25, P75)	61.0 (52.0–70.0)	62.0 (53.0–72.0)	59.5 (52.0–67.0)	0.006
AST, U/L, M (P25, P75)	22 (16–33)	22 (17–34)	20 (15–28)	0.005
ALT, U/L, M (P25, P75)	18 (12–30)	18 (12–32)	18 (11–30)	0.479
PT, s, M (P25, P75)	11.6 (11.0–12.2)	11.5 (10.9–12.1)	11.6 (11.1–12.5)	0.006
APTT, s, M (P25, P75)	25.9 (23.6–29.2)	25.7 (23.4–28.8)	27.1 (24.4–30.3)	0.002
Duration of surgery, h, M (P25, P75)	7.00 (5.50–8.50)	6.50 (5.30–8.30)	7.50 (6.50–8.85)	<0.001
Blood loss, mL, M (P25, P75)	100 (50–300)	100 (78–300)	100 (50–200)	0.002
SpO_2_, %, M (P25, P75)	100 (99–100)	100 (99–100)	100 (99–100)	0.026
Crystalloid, mL, M (P25, P75)	1500 (1500–2265)	2000 (1500–2500)	1500 (1000–2000)	<0.001
Colloid, mL, M (P25, P75)	1000 (500–1000)	1000 (500–1000)	500 (500–1000)	0.004
Fluid balance volume, mL/(kg·h), M (P25, P75)	5.56 (4.00–8.06)	6.45 (4.43–9.18)	4.56 (3.23–5.82)	<0.001
Urine output, mL, M (P25, P75)	1170 (800–1500)	1200 (800–1500)	1170 (700–1500)	0.129
PRBCs, U, M (P25, P75)	0 (0–2)	0 (0–2)	0 (0–2)	0.222
Plasma, mL, M (P25, P75)	0 (0–180)	0 (0–200)	0 (0–35)	0.415
Diuretics, *n* (%)	10 (1.76)	10 (2.49)	0 (0.00)	0.089
NSAIDs, *n* (%)	224 (39.51)	143 (35.57)	81 (49.09)	1.000
Glucocorticoids, *n* (%)	254 (44.72)	166 (41.29)	88 (53.01)	0.011
Vasoactive drugs, *n* (%)	8 (1.41)	6 (1.49)	2 (1.20)	1.000
Postoperative analgesia, *n* (%)	540 (95.07)	379 (94.28)	161 (96.99)	0.175
Postoperative analgesic effect				0.007
No pain, *n* (%)	19 (3.35)	15 (3.73)	4 (2.41)	
Mild to moderate pain, *n* (%)	275 (48.42)	208 (51.74)	67 (40.36)	
Severe pain, *n* (%)	274 (48.24)	179 (44.53)	95 (57.23)	

SBP, systolic blood pressure; DBP, diastolic blood pressure; ASA PS, American Society of Anesthesiologists Physical Status; ALB, albumin; AST, aspartate aminotransferase; ALT, alanine aminotransferase; PT, prothrombin time; APTT, activated partial thromboplastin time; SpO_2_, peripheral oxygen saturation; PRBCs, packed red blood cells; NSAID, nonsteroidal anti-inflammatory agent. Continuous data are presented as median (M) (interquartile range); categorical data are presented as *n* (%).

In this study, 63 patients developed vascular crisis. Among them, 37 patients (58.7%) underwent surgical exploration. Exploratory findings confirmed arterial crisis in 22 patients and venous crisis in 15 patients. Of the 26 patients who did not undergo surgical exploration, 24 presented with clinical manifestations consistent with mild venous crisis and showed significant clinical improvement following conservative management, including suture removal for tension relief, incision and bloodletting for decompression, and volume expansion. Therefore, surgical exploration was not performed, and the flaps ultimately survived. If conservative treatment had been ineffective, surgical exploration would have been performed. The overall salvage success rate among free flaps that developed vascular crisis was 73.0% (46/63). Among the 37 patients who underwent surgical exploration, 22 were successfully salvaged (59.5%). The remaining 2 patients with suspected vascular crisis were identified at a late stage, when the opportunity for surgical exploration had been missed; both flaps eventually became necrotic. The diagnosis in all patients strictly followed the clinical diagnostic criteria and was confirmed by two senior physicians.

Of the 568 patients included, 63 (11.1%) developed postoperative vascular crisis. Seventeen patients experienced flap necrosis, with an overall flap survival rate of 97.0%. The training set comprised 402 patients, of whom 42 (10.4%) experienced postoperative vascular crises. Fifteen patients experienced flap failure, with a free flap survival rate of 96.2%. The validation set comprised 166 patients, of whom 21 (12.7%) had postoperative vascular crises. Of these, two patients experienced flap failure, and the flap survival rate was 98.7%. Univariate screening was performed to detect the predictors of vascular crisis in free flaps. Using *P* < 0.10 as the screening threshold, 13 variables were finally retained for predictive modeling, whereas the remaining 30 were excluded for not meeting this criterion. Table 2 presents a comparison of the demographic and clinical characteristics of the participants in the training set with and without postoperative vascular crises.

**Table 2:** Demographic and clinical variables of patients with and without vascular crisis in the training set.

Variables	Vascular crisis group (*n* = 42)	Non-vascular crisis group (*n* = 360)	*p*-value
Age, years, M (P25, P75)	41.5 (25.5–49)	43 (29–49)	0.672
Sex (Male), *n* (%)	34 (80.95)	290 (80.56)	0.951
Weight, kg, M (P25, P75)	63.5 (59.1–75)	65 (60–75)	0.719
Pulse, beats per min, M (P25, P75)	80 (75–90)	80 (72–84)	0.805
Respiration, breaths per min, M (P25, P75)	16 (16–18)	16 (16–18)	0.834
SBP, mmHg, M (P25, P75)	129 (120–139)	128 (120–135)	0.666
DBP, mmHg, M (P25, P75)	80 (70–89)	80 (71–85)	0.669
Temperature, C, M (P25, P75)	36.5 (36.5–36.7)	36.6 (36.5–36.7)	0.558
ASA PS, *n* (%)			0.312
I	21 (50.00)	226 (62.78)	
II	21 (50.00)	121 (33.61)	
III	0 (0.00)	13 (3.61)	
Peripheral vascular disease, *n* (%)	0 (0.00)	0 (0.00)	1.000
Hypertension, *n* (%)	0 (0.00)	7 (1.94)	1.000
Cardiovascular diseases, *n* (%)	0 (0.00)	2 (0.56)	1.000
Diabetes, *n* (%)	0 (0.00)	5 (1.39)	1.000
Chronic kidney disease, *n* (%)	0 (0.00)	0 (0.00)	1.000
Pulmonary diseases, *n* (%)	0 (0.00)	1 (0.28)	1.000
Liver diseases, *n* (%)	0 (0.00)	2 (0.56)	1.000
Smoking, *n* (%)	28 (66.67)	137 (38.06)	<0.001
Alcohol, *n* (%)	8 (19.05)	125 (34.76)	0.041
Hemoglobin, g/ L, M (P25, P75)	110 (94–126)	130 (111–145)	<0.001
Hematocrit, %, M (P25, P75)	35.0 (28.1–41.3)	38.4 (32.4–42.5)	0.046
Platelets, 10⁹/L, M (P25, P75)	250 (210–314)	248 (205–310)	0.793
ALB, g/L, M (P25, P75)	33.35 (28.78–37.00)	37.60 (33.30–41.60)	<0.001
Fasting blood glucose, mmol/L, M (P25, P75)	5.2 (4.7–6.3)	5.2 (4.7–5.9)	0.957
Blood urea, mmol/L, M (P25, P75)	4.3 (3.2–5.0)	4.4 (3.5–5.5)	0.174
Creatinine, μmol/L, M (P25, P75)	64 (55–70)	62 (53–72)	0.890
AST, U/L, M (P25, P75)	28.0 (19.8–57.5)	22.0 (16.3–34.0)	0.020
ALT, U/L, M (P25, P75)	20.5 (12.0–38.6)	18.0 (12.0–32.0)	0.268
PT, s, M (P25, P75)	11.9 (11.1–12.7)	11.5 (10.9–12.1)	0.041
APTT, s, M (P25, P75)	25.8 (23.7–30.6)	25.7 (23.3–28.7)	0.561
Duration of surgery, h, M (P25, P75)	7.50 (6.35–9.00)	6.50 (5.16–8.00)	0.004
Blood loss, mL, M (P25, P75)	200 (138–500)	100 (50–300)	<0.001
SpO_2_, %, M (P25, P75)	99 (98–100)	100 (99–100)	0.001
Crystalloid, mL, M (P25, P75)	2000 (1500–2500)	2000 (1500–2500)	0.219
Colloid, mL, M (P25, P75)	1000 (500–1125)	1000 (500–1000)	0.068
Fluid balance volume, mL/(kg·h), M (P25, P75)	7.13 (4.46–8.93)	6.36 (4.31–8.46)	0.895
Urine output, mL, M (P25, P75)	1272 (1000–1700)	1200 (800–1500)	0.216
PRBCs, U, M (P25, P75)	2 (0–5)	0 (0–2)	<0.001
Plasma, mL, M (P25, P75)	0 (0–408)	0 (0–160)	0.004
Diuretics, *n* (%)	2 (4.76)	8 (2.22)	0.634
NSAIDs, *n* (%)	13 (30.95)	130 (36.11)	1.000
Glucocorticoids, *n* (%)	20 (47.62)	146 (40.56)	0.379
Vasoactive drugs, *n* (%)	0 (0.00)	6 (1.67)	1.000
Postoperative analgesia, *n* (%)	38 (90.48)	341 (94.72)	0.441
Postoperative analgesic effect			0.010
No pain, *n* (%)	2 (4.76)	13 (3.61)	
Mild to moderate pain, *n* (%)	12 (28.57)	196 (54.44)	
Severe pain, *n* (%)	28 (66.67)	151 (41.94)	

SBP, systolic blood pressure; DBP, diastolic blood pressure; ASA PS, American Society of Anesthesiologists Physical Status; ALB, albumin; AST, aspartate aminotransferase; ALT, alanine aminotransferase; PT, prothrombin time; APTT, activated partial thromboplastin time; SpO_2_, peripheral oxygen saturation; PRBCs, packed red blood cells; NSAID, nonsteroidal anti-inflammatory agent. Continuous data are presented as median (M) (interquartile range); categorical data are presented as *n* (%).

### 3.2 Missing data

Among the 568 patients, 70 (12.3%) had at least one missing value. Missing values were observed for intraoperative urine output and five preoperative laboratory variables: blood glucose, creatinine, urea, AST, and ALT. All other candidate predictors and the outcome variables were complete. The detailed extent and pattern of missing data are summarized in Table 3.

**Table 3:** Missing values in the training and validation cohorts.

Variable	Total (*N* = 568)	Training cohort (*n* = 402)	Validation cohort (*n* = 166)
Glucose	25 (4.4)	12 (3.0)	13 (7.8)
Urea	22 (3.9)	9 (2.2)	13 (7.8)
Creatinine	22 (3.9)	9 (2.2)	13 (7.8)
AST	22 (3.9)	9 (2.2)	13 (7.8)
ALT	22 (3.9)	9 (2.2)	13 (7.8)
Urine output	48 (8.5)	32 (8.0)	16 (9.6)

Data are presented as *n* (%). Percentages were calculated using the number of patients in each cohort as the denominator. Missing values were defined as unavailable results for the corresponding liver or kidney function laboratory test. Because a patient could have missing values for more than one parameter, the counts for individual variables may overlap and should not be summed. AST, aspartate aminotransferase; ALT, alanine aminotransferase.

Missing data were primarily attributable to the unavailability of preoperative liver and kidney function test results in the retrospective medical records. Specifically, in 22 patients, liver and kidney function test results were unavailable, resulting in simultaneous missingness for all five laboratory variables. In three patients, liver and kidney function tests were performed but blood glucose was not measured, resulting in missing glucose values. Urine output data were missing for 48 patients.

### 3.3 Flap types

The distribution of free flap types and postoperative vascular crisis are provided in Table 4. The three most common free flap types were the free anterolateral thigh flap (196, 34.5%), free forearm flap (155, 27.3%), and free thoracoumbilical flap (75, 13.2%). The remaining free flap types each accounted for ≤5.1% of all free flaps. The free anterolateral thigh flap had the highest absolute numbers of vascular crisis (27) and necrosis (8). No vascular crisis or necrosis was observed among free posterior tibial artery flaps or other free flaps.

**Table 4:** Distribution of free flap types and postoperative vascular crisis and necrosis in the training and validation cohorts, ordered by total number of flaps.

Flap type	Total (*N* = 568)	Overall proportion (%)	Training total (*n* = 402)	Training crisis	Training necrosis	Validation total (*n* = 166)	Validation crisis	Validation necrosis
Free anterolateral thigh flap	196	34.5	127	18	6	69	9	2
Free forearm flap	155	27.3	108	7	0	47	4	0
Free thoracoumbilical flap	75	13.2	71	7	4	4	1	0
Free gastrocnemius flap	29	5.1	22	4	1	7	2	0
Free foot flap	28	4.9	19	1	0	9	0	0
Free ilioinguinal flap	23	4.0	9	1	0	14	5	0
Free medial calf flap	20	3.5	17	2	2	3	0	0
Free posterior interosseous flap	18	3.2	11	1	1	7	0	0
Free latissimus dorsi flap	13	2.3	8	1	1	5	0	0
Free posterior tibial artery flap	9	1.6	8	0	0	1	0	0
Other free flaps	2	0.4	2	0	0	0	0	0
Total	568	100.0	402	42	15	166	21	2

Data are presented as *n* unless otherwise indicated. All flaps were free flaps; therefore, “Free” is prefixed to each flap type. Overall proportion (%) was calculated as the number of each free flap type divided by the total sample size (*N* = 568) and is reported to one decimal place. Rank was based on the total number of flaps of each type in descending order. Crisis, vascular crisis; necrosis, flap necrosis. Crisis and necrosis may coexist in the same flap or patient; therefore, the counts are not mutually exclusive. “Other free flaps” includes rare free flap types.

### 3.4 Development of the nomogram

A predictive model for estimating the risk of vascular crisis following free flap procedures was constructed based on data from 402 patients in the training set. According to the multivariate logistic regression findings, ALB level, smoking, Hb level, and PRBC transfusion were independent risk factors for vascular crisis after free flap surgery (Table 5). A predictive model for vascular crisis was established, and a nomogram was plotted based on these four predictors (Figure 2).

**Table 5:** Multivariate logistic regression analysis of independent risk factors for free flap vascular crisis.

Intercept and variables	*Β*	OR (95% CI)	SE	*p*-value
Intercept	2.982	—	—	—
ALB, g/L	−0.076	0.927 (0.868–0.991)	0.034	0.025
Smoker, yes vs. no	1.550	4.713 (2.184–10.171)	0.392	<0.001
Hb, g/L	−0.030	0.971 (0.952–0.990)	0.010	0.003
PRBCs, U	0.157	1.170 (0.993–1.377)	0.083	0.060

*Β*, *β* coefficient; CI, confidence interval; OR, odds ratio; SE, standard error; ALB, albumin; Hb, hemoglobin; PRBCs, packed red blood cells.

**Figure 2:**
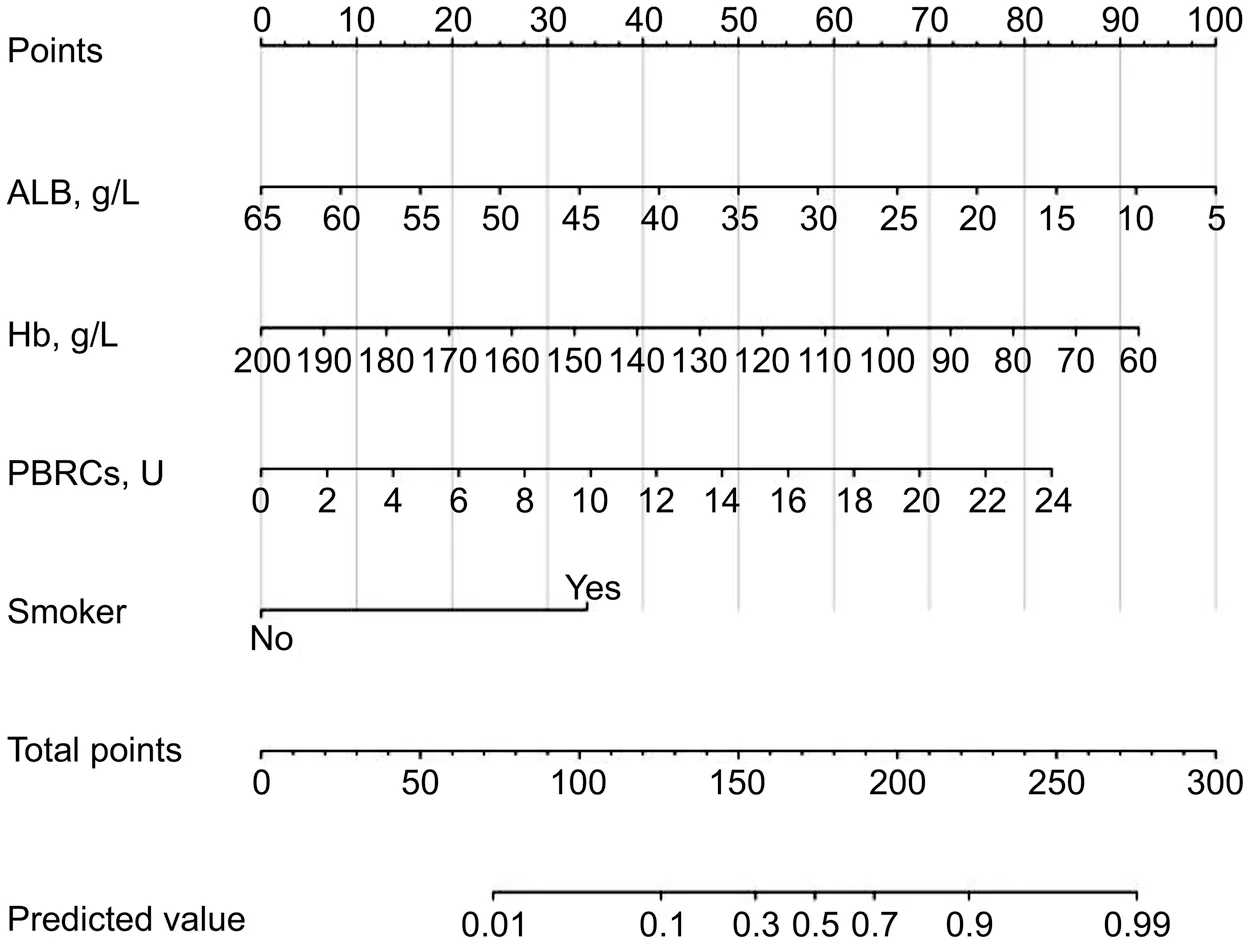
Nomogram to predict postoperative vascular crisis risk in patients receiving free flap surgery.

Procedure for using the nomogram to predict the risk of postoperative vascular crisis in free flaps:

**Step 1**: The corresponding positions of each predictor variable are determined based on their numerical values.
**Step 2**: The corresponding scores for each variable are recorded on the score axis at the top of the nomogram, and the score is computed by summing the points assigned to each predictive factor.
**Step 3:** Based on the cumulative score, the matching coordinate on the risk probability scale at the bottom of the nomogram is identified to determine the predicted probability of an individual experiencing a postoperative vascular crisis.

To improve clinical applicability, a dynamic online graphical tool was constructed (https://jianglin.shinyapps.io/dynnomapp/). Real-time risk assessment can be performed by inputting the four predictive variables, with the output including the predicted probability and 95% confidence interval (CI) (Figure 3).

**Figure 3:**
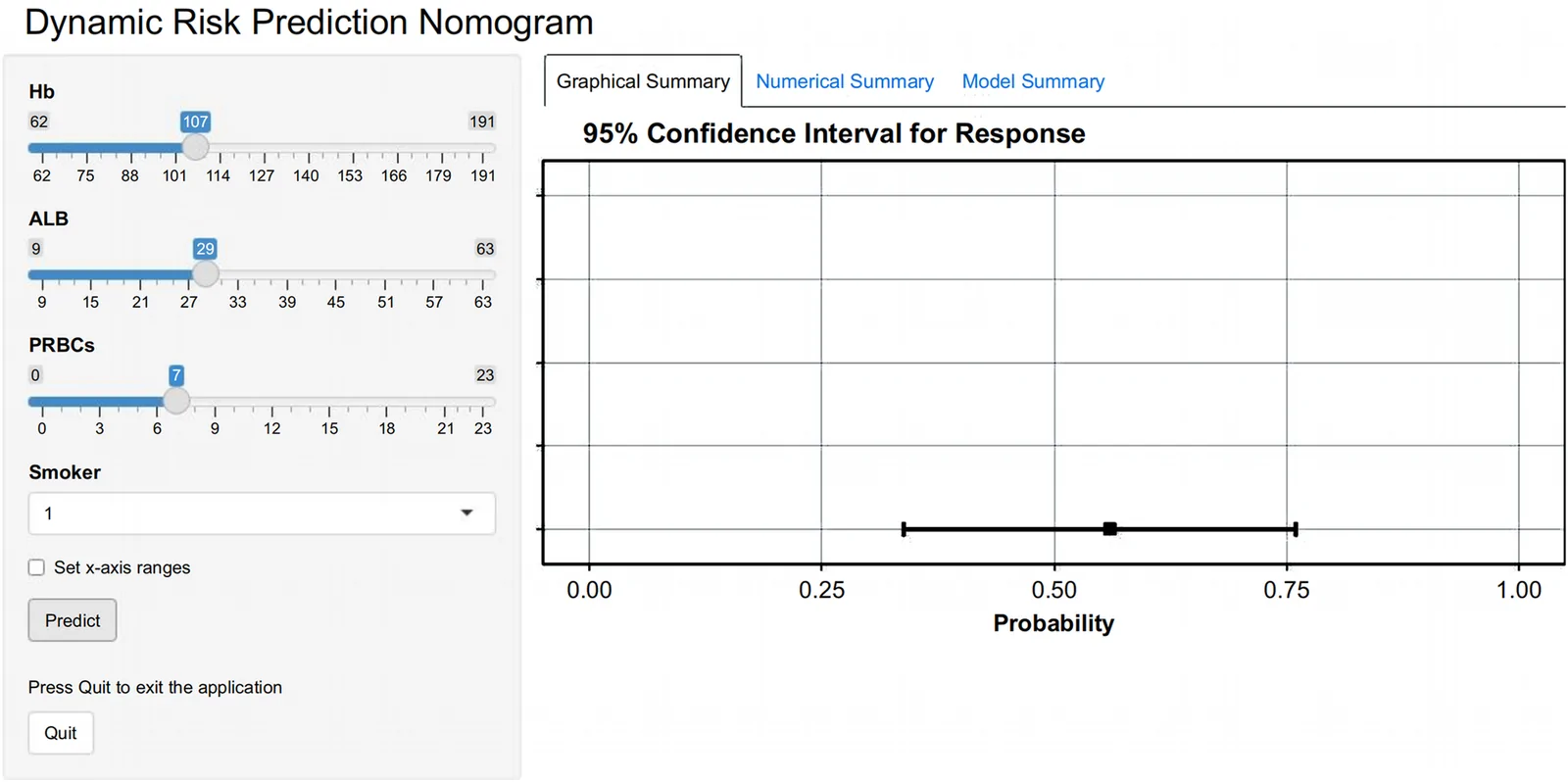
Dynamic web-based visualization calculator for free flap vascular crisis risk assessment.

### 3.5 Validation of the nomogram

The validation cohort consisted of 166 patients, and internal validation analyses were performed to assess the discriminative efficacy of the nomogram. The AUC with 95% CI was 0.817 (95% CI: 0.747–0.887) for the training cohort and 0.852 (95% CI: 0.774–0.929) for the validation cohort, indicating an appropriate predictive ability for free flap vascular crisis (Figure 4). The calibration curve closely approximated the ideal curve, showing a strong agreement between the actual and predicted outcomes (Figure 5). Moreover, the mean absolute error was 0.019, corresponding to an average prediction error of only 1.9%, which suggests that the model was highly accurate for calibration. According to the DCA, the prediction model offered significant net clinical utility across a risk threshold interval of 1% to 51% (Figure 6) (26–28). In addition, a complete-case analysis was performed as a sensitivity analysis, and the results were consistent with the primary analysis, supporting the robustness of our findings.

**Figure 4:**
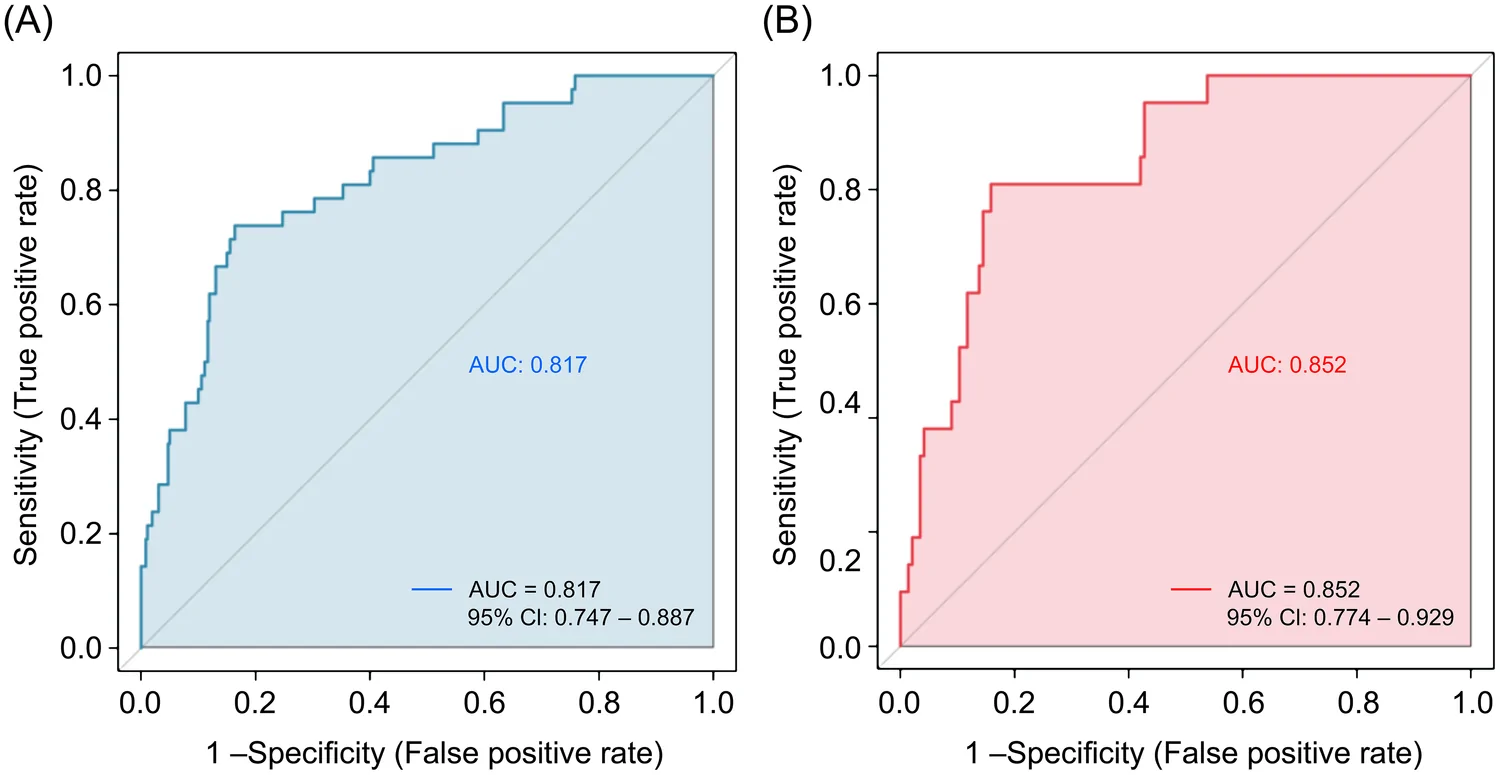
ROC curves of the nomogram for predicting free flap vascular crisis. **(A)** Training set; **(B)** Validation set.

**Figure 5:**
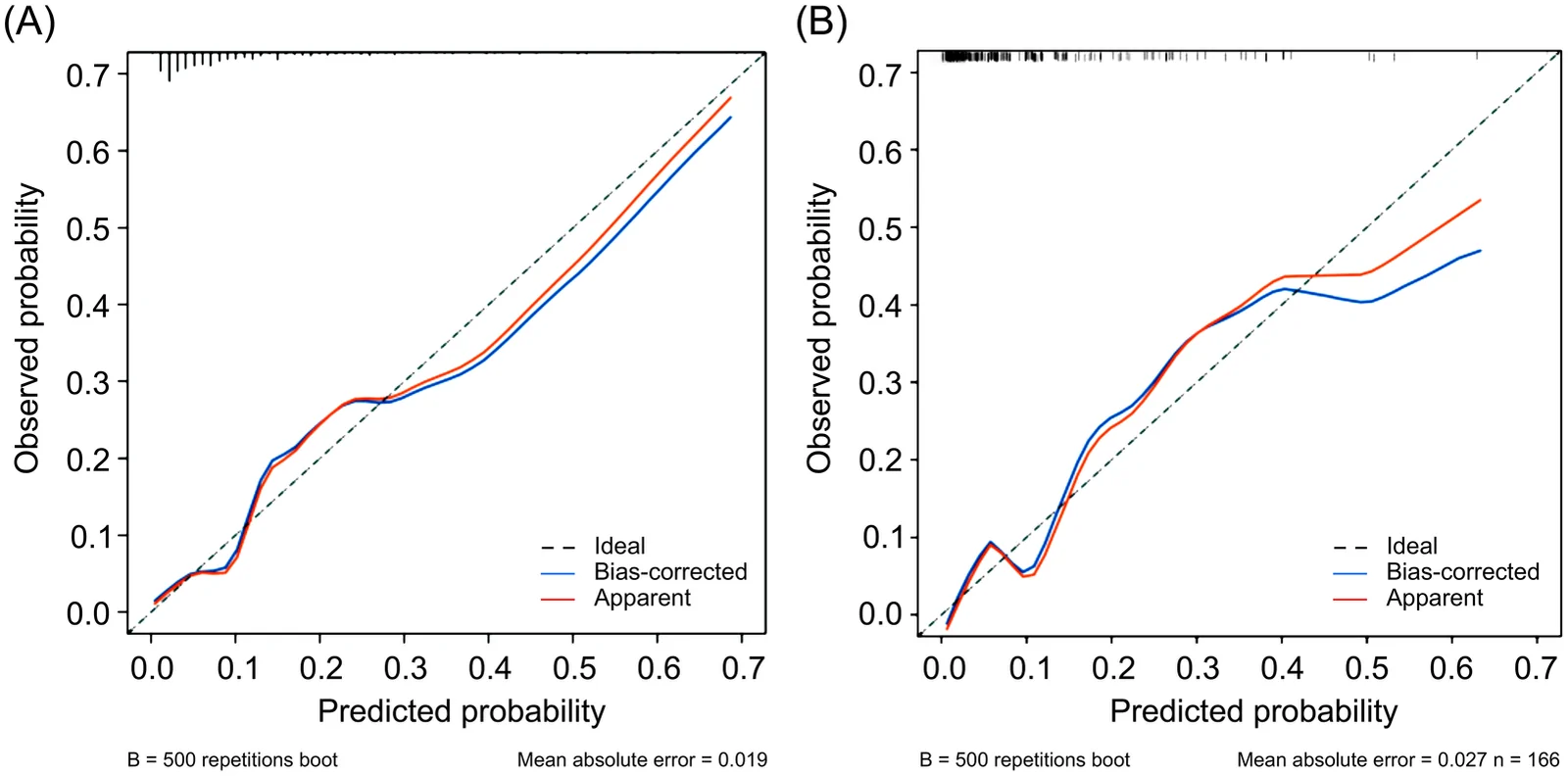
Calibration curve of the nomogram for predicting free flap vascular crisis. **(A)** Training set; **(B)** Validation set.

**Figure 6:**
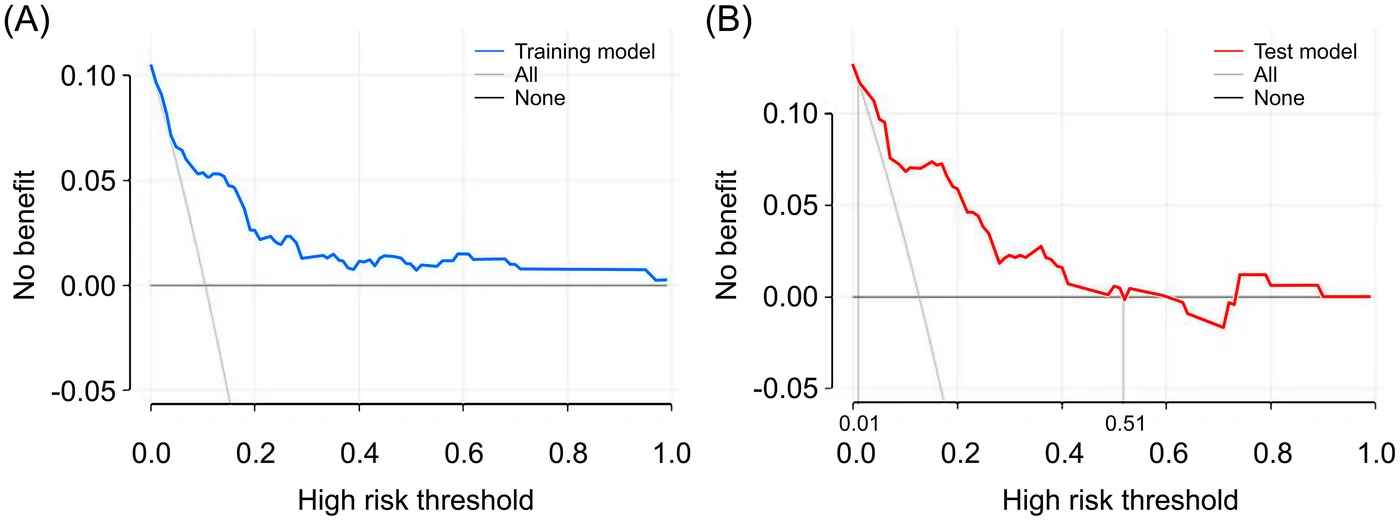
DAC curves of the nomogram for predicting free flap vascular crisis. **(A)** Training set; **(B)** Validation set.

## 4 Discussion

In this study, among 568 patients who underwent free flap surgery, 63 developed postoperative vascular crises, yielding an incidence of 11.1%. Seventeen flaps failed to survive, corresponding to a success rate of 97.0%. Four independent risk factors were identified: serum ALB level, smoking status, Hb level, and PRBC transfusion status. A nomogram for predicting free flap vascular crisis was constructed based on the training cohort, and internal validation was performed using the validation sample, accompanied by further model visualization. The predictive model achieved AUC values of 0.817 and 0.852 in the training and validation cohorts, respectively. Favorable concordance was observed in the calibration curve, and DCA indicated a clear clinical benefit. This novel nomogram prediction model exhibited good discriminatory performance for predicting the risk of free flap vascular crisis.

It should be noted that the diagnosis of vascular crisis in this study was primarily based on clinical parameters rather than requiring surgical exploration in all cases. This approach reflects real-world clinical practice, in which early detection and timely conservative intervention may successfully reverse mild vascular compromise without the need for surgical exploration (29). Indeed, the difference between the number of vascular crises (*n* = 63) and the number of flap losses (*n* = 17) in our cohort underscores the effectiveness of early diagnosis and aggressive salvage efforts. Yang et al. demonstrated that the salvage rate for free flap crises detected within 8 h postoperatively was 66.7%, compared with only 22.2% for those detected after 48 h (30). Our salvage rate of 73.0% is comparable to these published figures, supporting the validity of our diagnostic and management approach.

In previous studies, the success rate of free flap surgery generally exceeded 95%, reaching > 98% in experienced medical centers (31, 32). Nevertheless, the medical community generally accepts the inherent risk of failure, because free flap surgery remains irreplaceable. In the fields of traumatic limb salvage and head and neck oncological functional reconstruction, the benefits of free flaps far outweigh the risks of potential flap failure. The salvage success rate in this investigation was 73%, which is in accordance with the data from previous studies (2). Postoperative vascular crisis is the primary cause of free flap failure and represents a major postoperative risk factor (25). Effective prevention of vascular crises is critical for ensuring the success of free flap surgery, alleviating patient suffering, avoiding salvage surgery, and shortening the length of hospital stay (33). Prevention of vascular crises during intraoperative management, postoperative planning, and postoperative care relies on the identification of high-risk individuals (34). Furthermore, variations in risk factors serve as the key basis for adopting individualized prevention strategies.

In this study, the independent risk factors collectively pointed to the patient's general condition, oxygen supply capacity, and microcirculatory environment, suggesting that vascular crisis is determined not only by surgical capability, but also by systemic status and perioperative stress response (35). This finding is consistent with the known causes of free flap failure. This aligns with the etiology of free flap failure; in addition to surgical factors, systemic factors such as systemic hypovolemia and microcirculatory dysfunction significantly contribute to thrombosis (36, 37). The four independent risk factors demonstrated clear clinical and pathophysiological associations.

Low serum ALB levels indicate a poor perioperative nutritional status. Nutritional status is a crucial factor for recovery after free flap transplantation, and malnutrition frequently results from high perioperative consumption during the perioperative period. Patients with trauma undergoing multiple surgeries may experience rapid weight loss, and malnourished individuals exhibited a 3.37-fold increased likelihood of free flap failure compared to well-nourished patients (38). Traumatic exudation and inflammatory reactions are the primary causes of hypoalbuminemia in patients with trauma and contribute to a significantly increased risk of thrombosis through various interconnected pathways. Evidence demonstrates that hypoalbuminemia accelerates thrombus formation by increasing fibrin clot density, inhibiting fibrinolysis, reducing platelet inhibition, and inducing a hypercoagulable state. Hypoalbuminemia decreases colloid osmotic pressure and alters cell membrane permeability, leading to tissue edema and microcirculatory dysfunction, which impair tissue healing and microcirculatory stability.

Smoking significantly contributes to vascular endothelial damage, vasospasm, and microthrombosis (39). Nicotine and other constituents of tobacco stimulate the sympathetic nervous system, resulting in substantial secretion of catecholamines that induce intense contraction of small arteries in the microvasculature, resulting in sluggish blood flow and stasis (40). Research indicates that in experimental smoking models, arterial perfusion may decline to 72% of normal levels (41). Furthermore, long-term smoking not only directly damages vascular endothelial cells, but also reduces nitric oxide levels and upregulates inflammatory factors due to the high levels of oxidants and free radicals in tobacco, thereby significantly delaying vascular endothelial repair (42, 43).

Hb content determines the oxygen-carrying capacity of blood, and studies have shown that both preoperative Hb levels and perioperative Hb fluctuations are independent predictors of vascular crisis in free flaps. In cases of anemia, insufficient local oxygen supply to the free flap may induce ischemic-reperfusion injury, thereby increasing the risk of vascular crisis (38). The mechanism underlying the vascular crisis associated with Hb remains debated and involves an interplay between prothrombotic and compensatory protective effects. First, mild anemia and ischemia in free flaps do not induce systemic hemodynamic redistribution. In patients with small-diameter flap vessels and preexisting ischemic risk, methemoglobin may enhance oxygen supply under limited flow and partially compensate for low perfusion. However, moderate-to-severe anemia triggers systemic hemorrhagic compensation with hemodynamic redistribution to preserve vital organ perfusion. Because free flaps are peripheral and nonvital organs, their blood supply is further reduced during redistribution. Although Hb is associated with blood viscosity, it remains unclear whether low Hb levels improve vascular patency in free flaps. Notably, patients undergoing free flap surgery differ from those with typical trauma (44). Perioperative circulatory management should not be limited to stabilizing vital signs but should optimize systemic perfusion and oxygenation to restore near-physiological conditions and improve flap oxygenation and support (4).

PRBC transfusion is used to improve blood oxygen-carrying capacity. In clinical practice, transfusions are administered to correct high-risk anemia rather than as proactive prophylaxis against vascular crises. Therefore, PRBC volume indirectly reflects the severity of intraoperative blood loss or preoperative anemia. Currently, no uniform Hb threshold has been established for initiating blood transfusions or defining transfusion targets during free flap procedures. The objective of intraoperative transfusion is to maintain circulation with the minimum possible blood volume; therefore, transfusion serves as an indicator of a compromised circulatory status. However, intraoperative anesthetic management following routine volume-controlled strategies that maintain circulatory function at a low flow level may not adequately meet the oxygen-carrying requirements of the free flap (25). Transfusion inevitably leads to reduced nitric oxide levels, enhanced endogenous coagulation, vascular endothelial damage, and inflammatory activation (45). Animal studies have demonstrated that perioperative hemodilution is superior to blood transfusion during free flap surgery (46). As free flaps represent the terminal segment of the peripheral circulation, their circulatory and metabolic characteristics differ substantially from those of vital organs. The limited improvement in microcirculatory perfusion following transfusion may also be a critical contributor to vascular crisis.

Previous surveys have indicated that microsurgeons and hematologists have different understandings of the risk factors for free flap failure (47). Surgeons consider operative duration, number of vascular anastomoses, history of arterial thrombosis, and predisposition to thrombosis to be the most significant risk factors. In contrast, hematologists emphasize the importance of diabetes mellitus, smoking, and other comorbidities. Understanding these variations in risk assessment from different perspectives can accelerate the multidimensional analysis of variables and standardize the development of risk prediction models for vascular crises. The core objective is to achieve precise risk stratification at the end of surgery for populations at high-risk of vascular crises and optimize perioperative management strategies. This study used classic logistic regression to construct a predictive risk model based on perioperative management by anesthetists (28). First, the predictive model demonstrates a clear advantage in terms of variable controllability. Compared with non-modifiable factors such as obesity, diabetes, hypercoagulability, recipient vessel condition, number of anastomoses, and catecholamines, the three predictive variables (serum ALB, Hb, and PRBC transfusion) are directly modifiable, whereas smoking can be effectively managed, thereby making intervention measures highly feasible. Second, the model exhibits good clinical applicability. The output results are a visual nomogram with an online calculator, which enables the medical staff to instantly assess risks using mobile phones without the need for dedicated computing equipment, rendering it suitable for widespread application. Third, the model has strong clinical interpretability. The regression coefficients quantify the impact of each risk factor and clarify the independent weights of each variable. Unlike the ‘black-box’ nature of machine learning algorithms, including ensemble decision trees and artificial neural networks, this model is more easily understood and accepted by clinicians, facilitating result interpretation combined with clinical experience and guiding intervention decisions (48, 49). Compared with other similar models, the present nomogram shifts the focus of vascular crisis prevention from surgical procedures to perioperative status management, uses systemic optimization as the key to risk prevention, and offers a useful adjunct to existing prediction tools (50).

However, this study has certain limitations. First, its retrospective design was inherently biased in nature. Information was obtained from a single-center electronic medical record database and may have been subject to selection and information biases (e.g., unrecorded or unmeasured confounding factors). This is a common issue in retrospective studies. Similarly, in their single-center study of free head and neck flaps, Suzuki et al. observed that the limitations of single-center data may affect the generalizability of the results (5). Second, the range of predictive factors should be expanded. The model included only four variables and did not evaluate surgical quality. Although this approach is simple and practical, it may overlook other important factors (e.g., the specific surgical site and number of anastomosed vessels). In the future, multidisciplinary variables, such as those related to surgery and intensive care, should be incorporated to further optimize the model. Third, external validation was not performed. To date, only internal validation has been conducted. The performance of the model in other centers and among different populations requires further confirmation through multicenter external validation. Multicenter prospective studies are essential to enhance the reliability and generalizability of predictive models. Existing research has consistently emphasized the importance of external validation.

## 5 Conclusion

The nomogram-based risk prediction model constructed in this study was validated and demonstrated satisfactory predictive performance. The predictive variables of this model provide important clinical guidance for the management of perioperative general conditions. Based on these variables, surgeons are advised to enhance the quality of surgery, increase nutritional intake, improve overall physical condition, strengthen smoking cessation education, and enforce ward smoking bans as important strategies for perioperative risk prevention. To treat anemia with PRBC transfusion, initiating transfusion as early as possible before surgery, appropriately postponing the procedure, or adopting a restrictive transfusion strategy during surgery may be more effective in minimizing the impact of transfusion and maintaining stable peripheral terminal microcirculation in free flaps.

## Data Availability

The raw data supporting the conclusions of this article will be made available by the authors, without undue reservation.
