# Supplementary material for: Construction and validation of a predictive nomogram for vascular crisis risk following free flap surgery
Source: Front Med (Lausanne). 2026 Oct 20;13:1930369. doi: 10.3389/fmed.2026.1930369 (PMC13727465; doi:10.3389/fmed.2026.1930369)
Supplement: Supplementary file 1 [file Table1.docx]

STROBE Statement—checklist of items that should be included in reports of observational studies

|  | | Item No | | Recommendation | Page  No | |
| --- | --- | --- | --- | --- | --- | --- |
| **Title and abstract** | | 1 | | (*a*) Indicate the study’s design with a commonly used term in the title or the abstract | Page 1. Line 1. Lines 24-29. | |
|  |  |  |  | (*b*) Provide in the abstract an informative and balanced summary of what was done and what was found | Page 1-2. Lines 32-42. | |
| Introduction | | | | | | |
| Background/rationale | | 2 | | Explain the scientific background and rationale for the investigation being reported | Page 2-3, Introduction  Section. | |
| Objectives | | 3 | | State specific objectives, including any prespecified hypotheses | Page 3. Lines 89-94. | |
| Methods | | | | | | |
| Study design | | 4 | | Present key elements of study design early in the paper | Page 3, Materials and methods, Study population. Lines 89–100. | |
| Setting | | 5 | | Describe the setting, locations, and relevant dates, including periods of recruitment, exposure, follow-up, and data collection | Page 3. Lines 97-100. | |
| Participants | | 6 | | (*a*) *Cohort study*—Give the eligibility criteria, and the sources and methods of selection of participants. Describe methods of follow-up | Page 3. Lines 110-116. Page 5. Lines 185-189.  Figure 1 . | |
| Variables | | 7 | | Clearly define all outcomes, exposures, predictors, potential confounders, and effect modifiers. Give diagnostic criteria, if applicable | Page 4, outcome measure and data collection section. Lines 117-154. | |
| Data sources/ measurement | | 8* | | For each variable of interest, give sources of data and details of methods of assessment (measurement). Describe comparability of assessment methods if there is more than one group | Page 4-5. Lines 130-167. | |
| Bias | | 9 | | Describe any efforts to address potential sources of bias | Page 4. Lines 126-128. Lines 133-134. Page 5. Lines 169-178. | |
| Study size | | 10 | | Explain how the study size was arrived at | Page 5. Lines 193-195.  Figure 1 | |
| Quantitative variables | | 11 | | Explain how quantitative variables were handled in the analyses. If applicable, describe which groupings were chosen and why | Page 5, Lines 180-184. | |
| Statistical methods | | 12 | | (*a*) Describe all statistical methods, including those used to control for confounding | Page 5-6, Statistical analysis section. | |
|  |  |  |  | (*b*) Describe any methods used to examine subgroups and interactions | Not Available. | |
|  |  |  |  | (*c*) Explain how missing data were addressed | Page 5, Lines 169-178. | |
|  |  |  |  | (*d*) *Cohort study*—If applicable, explain how loss to follow-up was addressed  *Case-control study*—If applicable, explain how matching of cases and controls was addressed  *Cross-sectional study*—If applicable, describe analytical methods taking account of sampling strategy | Not Available. | |
|  |  |  |  | (*e*) Describe any sensitivity analyses | Page 5, Lines 177-178. | |
| Results | | | | | | |
| Participants | 13* | | (a) Report numbers of individuals at each stage of study—eg numbers potentially eligible, examined for eligibility, confirmed eligible, included in the study, completing follow-up, and analysed | | | Page 6, demographic and clinical characteristics. Lines 206-215.  Figure 1. |
|  |  |  | (b) Give reasons for non-participation at each stage | | | Page 6, Lines 210-213.  Figure 1. |
|  |  |  | (c) Consider use of a flow diagram | | | Figure 1 |
| Descriptive data | 14* | | (a) Give characteristics of study participants (eg demographic, clinical, social) and information on exposures and potential confounders | | | Page 6, Lines 214-219, 233-242, 254-259.  Table 1, Table 2and Table 4. |
|  |  |  | (b) Indicate number of participants with missing data for each variable of interest | | | Page 7, Lines 244-252. Table 3. |
|  |  |  | (c) *Cohort study*—Summarise follow-up time (eg, average and total amount) | | | Not Available. |
| Outcome data | 15* | | *Cohort study*—Report numbers of outcome events or summary measures over time | | | Page 6, Lines 213-237.  Table 2. Table 4. |
| Main results | 16 | | (*a*) Give unadjusted estimates and, if applicable, confounder-adjusted estimates and their precision (eg, 95% confidence interval). Make clear which confounders were adjusted for and why they were included | | | Page 7. Lines 261–265;  Table 2. Table 5. |
|  |  |  | (*b*) Report category boundaries when continuous variables were categorized | | | Not Available |
|  |  |  | (*c*) If relevant, consider translating estimates of relative risk into absolute risk for a meaningful time period | | | Page 7. Lines 261-277. |
| Other analyses | 17 | | Report other analyses done—eg analyses of subgroups and interactions, and sensitivity analyses | | | Page 8. Lines 284-289 . |
| Discussion | | | | | | |
| Key results | 18 | | Summarise key results with reference to study objectives | | | Page 8. Lines 291-300. |
| Limitations | 19 | | Discuss limitations of the study, taking into account sources of potential bias or imprecision. Discuss both direction and magnitude of any potential bias | | | Page 10-11. Lines 408-422. |
| Interpretation | 20 | | Give a cautious overall interpretation of results considering objectives, limitations, multiplicity of analyses, results from similar studies, and other relevant evidence | | | Page 8-11. Lines 291-432. |
| Generalisability | 21 | | Discuss the generalisability (external validity) of the study results | | | Page 10. Lines 404-407. Page 11. Lines 416-422. |
| Other information | | | | | | |
| Funding | 22 | | Give the source of funding and the role of the funders for the present study and, if applicable, for the original study on which the present article is based | | | Page11. Funding. |

*Give information separately for cases and controls in case-control studies and, if applicable, for exposed and unexposed groups in cohort and cross-sectional studies.

**Note:** An Explanation and Elaboration article discusses each checklist item and gives methodological background and published examples of transparent reporting. The STROBE checklist is best used in conjunction with this article (freely available on the Web sites of PLoS Medicine at http://www.plosmedicine.org/, Annals of Internal Medicine at http://www.annals.org/, and Epidemiology at http://www.epidem.com/). Information on the STROBE Initiative is available at www.strobe-statement.org.
