# Supplementary material for: Construction and validation of a predictive nomogram for vascular crisis risk following free flap surgery
Source: Front Med (Lausanne). 2026 Oct 20;13:1930369. doi: 10.3389/fmed.2026.1930369 (PMC13727465; doi:10.3389/fmed.2026.1930369)
Supplement: Supplementary file 2 [file Table2.docx]

| **Section/Topic** | **Item** | **Checklist Item** | **Page** |
| --- | --- | --- | --- |
| **Title and abstract** | | | |
| Title | 1 | Identify the study as developing and/or validating a multivariable prediction model, the target population, and the outcome to be predicted. | Page 1. Lines 1-2. |
| Abstract | 2 | Provide a summary of objectives, study design, setting, participants, sample size, predictors, outcome, statistical analysis, results, and conclusions. | Page 1-2. Lines 23-42. |
| **Introduction** | | | |
| Background and objectives | 3a | Explain the medical context (including whether diagnostic or prognostic) and rationale for developing or validating the multivariable prediction model, including references to existing models. | Page 2-3, Introduction.  Lines 46-88. |
|  | 3b | Specify the objectives, including whether the study describes the development or validation of the model or both. | Page 3, Lines 89-94. |
| **Methods** | | | |
| Source of data | 4a | Describe the study design or source of data (e.g., randomized trial, cohort, or registry data), separately for the development and validation data sets, if applicable. | Page 3, Lines 95-100.Page 4. Lines 130-138. Page 5. Lines 185-189. |
|  | 4b | Specify the key study dates, including start of accrual; end of accrual; and, if applicable, end of follow-up. | Page 3. Lines 97–100. Page 5. Lines 185–189. |
| Participants | 5a | Specify key elements of the study setting (e.g., primary care, secondary care, general population) including number and location of centres. | Page 3, Lines 97-100. |
|  | 5b | Describe eligibility criteria for participants. | Page 3, Inclusion and exclusion criteria. Lines 110–116. |
|  | 5c | Give details of treatments received, if relevant. | Page 4. Lines 155–159. Page 5. Lines 160–167. |
| Outcome | 6a | Clearly define the outcome that is predicted by the prediction model, including how and when assessed. | Page 4, Outcomes measures. Lines 118-128. |
|  | 6b | Report any actions to blind assessment of the outcome to be predicted. | Not Available. |
| Predictors | 7a | Clearly define all predictors used in developing or validating the multivariable prediction model, including how and when they were measured. | Page 4, Lines 130-153. |
|  | 7b | Report any actions to blind assessment of predictors for the outcome and other predictors. | Page 3, Lines 124-125. |
| Sample size | 8 | Explain how the study size was arrived at. | Page 5, Lines 193-195. |
| Missing data | 9 | Describe how missing data were handled (e.g., complete-case analysis, single imputation, multiple imputation) with details of any imputation method. | Page 5. Lines 169–178. Page 7. Lines 244–252; Table 3. |
| Statistical analysis methods | 10a | Describe how predictors were handled in the analyses. | Page 5, Lines 180-196. |
|  | 10b | Specify type of model, all model-building procedures (including any predictor selection), and method for internal validation. | Page 5-6, Lines 190-203. |
|  | 10d | Specify all measures used to assess model performance and, if relevant, to compare multiple models. | Page 6, Lines 197-203. |
| Risk groups | 11 | Provide details on how risk groups were created, if done. | Not Available. |
| **Results** | | | |
| Participants | 13a | Describe the flow of participants through the study, including the number of participants with and without the outcome and, if applicable, a summary of the follow-up time. A diagram may be helpful. | Page 6, Lines 206-214. Figure 1 |
|  | 13b | Describe the characteristics of the participants (basic demographics, clinical features, available predictors), including the number of participants with missing data for predictors and outcome. | Page 6. Lines 215–219, 233–242. Page 7. Lines 244–252; Table 1–3. |
| Model development | 14a | Specify the number of participants and outcome events in each analysis. | Page 6, Lines 213-215. Table 2. |
|  | 14b | If done, report the unadjusted association between each candidate predictor and outcome. | Page 6. Lines 233–242. Table 2. |
| Model specification | 15a | Present the full prediction model to allow predictions for individuals (i.e., all regression coefficients, and model intercept or baseline survival at a given time point). | Page 7. Lines 261–277. Table 5. Figure 2. |
|  | 15b | Explain how to the use the prediction model. | Page 7, Lines 266-277.  Figure 3. |
| Model performance | 16 | Report performance measures (with CIs) for the prediction model. | Page 8, Lines 279-289.  Figure 4-6. |
| **Discussion** | | | |
| Limitations | 18 | Discuss any limitations of the study (such as nonrepresentative sample, few events per predictor, missing data). | Page 10-11, Lines 408-422. |
| Interpretation | 19b | Give an overall interpretation of the results, considering objectives, limitations, and results from similar studies, and other relevant evidence. | Page8-10, Lines 301-407. |
| Implications | 20 | Discuss the potential clinical use of the model and implications for future research. | Page 10, Lines 382-407. Page 11, Lines 424-432. |
| **Other information** | | | |
| Supplementary information | 21 | Provide information about the availability of supplementary resources, such as study protocol, Web calculator, and data sets. | Page 3, Lines 106-108.  Page 16, Line 596. |
| Funding | 22 | Give the source of funding and the role of the funders for the present study. | Page 11, Funding. Lines 442-445. |

We recommend using the TRIPOD Checklist in conjunction with the TRIPOD Explanation and Elaboration document.
